# Preventing and Monitoring Work-Related Diseases in Firefighters: A Literature Review on Sensor-Based Systems and Future Perspectives in Robotic Devices

**DOI:** 10.3390/ijerph18189723

**Published:** 2021-09-15

**Authors:** Juri Taborri, Simone Pasinetti, Ludovica Cardinali, Fabrizio Perroni, Stefano Rossi

**Affiliations:** 1 Department of Economics, Engineering, Society and Business Organization, University of Tuscia, 01100 Viterbo, Italy; stefano.rossi@unitus.it; 2 Department of Mechanical and Industrial Engineering, University of Brescia, 25121 Brescia, Italy; simone.pasinetti@unibs.it; 3 Department of Movement, Human and Health Sciences, University of Rome “Foro Italico”, 00135 Rome, Italy; l.cardinali1@studenti.uniroma4.it; 4 Department of Biomolecular Sciences, Section of Exercise and Health Sciences, University of Urbino Carlo Bo, 61029 Urbino, Italy; fabrizio.perroni@uniurb.it

**Keywords:** firefighters, wearable sensors, robotic devices, work-related disease, physiological and physical parameters

## Abstract

In recent years, the necessity to prevent work-related diseases has led to the use of sensor-based systems to measure important features during working activities. This topic achieved great popularity especially in hazardous and demanding activities such as those required of firefighters. Among feasible sensor systems, wearable sensors revealed their advantages in terms of possibility to conduct measures in real conditions and without influencing the movements of workers. In addition, the advent of robotics can be also exploited in order to reduce work-related disorders. The present literature review aims at providing an overview of sensor-based systems used to monitor physiological and physical parameters in firefighters during real activities, as well as to offer ideas for understanding the potentialities of exoskeletons and assistive devices.

## 1. Introduction

Every day, firefighters (FFs) are involved in activities, which are both physically and psychologically demanding, to protect the safety and the well-being of the community [1]. An observational study lasting five years conducted by an American research groups showed that 368 of 1150 career firefighters died during emergency responses; in particular, 39% from heart attacks and the remaining 61% from other causes, such as myocardial infraction, asphyxia and motor vehicle crashes [2].

The high rate of cardiovascular disease and mortality in FFs mainly depends on the interaction with several factors that are typical of working activities, such as sympathetic activation [3,4], physical workload [5,6,7], heat [8,9,10], dehydration [8,11,12], incorrect physical activities [13,14], shift work [15,16,17,18], exposure to physical and chemical agents [19,20] and psychological stress [11,21,22,23,24]. In addition, in emergency situations, the combination of physical demands, unpredictable conditions, warm clothing and exposure to external sources of risk leads to an increase in physiological and psychological stress, which can lead to impaired cognitive functioning, also causing risks for the community [3,4,5,11,25,26]. Several studies have then demonstrated the deterioration of physical abilities due to ageing [27,28]; however, such an effect is drastically reduced in FFs with a lower body mass index [27]. Thus, the implementation of specific training programs is mandatory for both the increment and the maintaining of physical performance. As a further risk factor for the occurrence of work-related diseases, the personal protective equipment (PPE) that FFs have to wear during their activities play an influencing role. In fact, PPE consist of protective clothes with several layers, heavy footwear, a helmet and self-contained breathing apparatus, also known as SCBA. The use of PPE has been correlated with a significant increase in injury risk [29]. Considering the physiological effects, a reduction up to 20% of metabolic efficiency and thermoregulation and up to 75% of work tolerance has been found due to the presence of the PPE [12,30,31,32,33,34,35]. Considering the biomechanics, the effects of PPE on joint angles, spatio-temporal parameters and postural variables have been demonstrated. In this context, it emerges that monitoring physiological and physical parameters during FF activities can allow us to obtain useful information in order to adopt recommendations regarding fitness standards, mandatory medical evaluations and to optimize the design of PPE, with the final aim to prevent or reduce the work-related disorders.

For all the above-mentioned reasons, several studies deal with the analysis of physiological variables [6,36,37,38,39,40,41,42,43,44,45,46,47,48,49,50,51,52,53,54,55,56,57,58,59,60,61,62,63,64,65,66,67,68,69,70,71,72,73,74,75,76,77,78,79,80,81,82,83,84,85,86,87] and physical parameters [88,89,90,91,92,93,94,95,96,97,98,99,100,101,102,103,104,105,106,107]. Sensor-based systems, such as portable metabolic systems, heart rate monitors, ingestible temperature pills and skin temperature patches for the physiological aspects [6,25,46,48,53,55,56,57,71,74,78,81,84], as well optoelectronic systems, inertial sensors and pressure insoles for the physical ones [94,95,96,97,98,101,102,108,109] have shown their potential mainly due to the possibility to measure several variables without influencing the FF working procedures. Moreover, the latest progress in the robotic field has opened the possibility to use robotic devices to help prevent worker fatigue and work-related musculo-skeletal disorders in manufacturing settings. Considering the type of movements performed daily by FFs and the long durations of their interventions, the design of specific exoskeletons or assisting robots for supporting firefighters during work is an attractive research field [110,111,112,113,114,115,116,117,118].

To the best of the authors’ knowledge, no review articles have been proposed focusing on the application of sensor-based systems and robotic devices in firefighters. Thus, the present review aims at providing an overview of how the sensor-based systems have been used during firefighters’ activities to monitor physiological and physical parameters to reduce disease risks, as well as an overview of robotic devices to support firefighters and prevent work-related disorders.

## 2. Literature Survey and Analysis

### 2.1. Search Strategy and Inclusion Criteria

The literature overview was conducted by means of Scopus, Web of Science and PubMed, focusing on the studies that describe the use of sensor-based systems and robotic devices among firefighters. In particular, two categories of sensors have been taken into account: those for physiological or physical measures, as well two categories of robotic devices, i.e., exoskeletons (for upper or lower limbs) or assisting devices. The literature review was performed from March to December 2020 and all the combinations of the following keywords were used: *sensor-based systems, wearable sensors, robotic devices, firefighters, work-related diseases, physiological parameters; kinematics; exoskeletons; personal protective equipment, portable device, real-time monitoring, robots.* Wildcard symbols were also taken into account to avoid forgetting important studies. The reference list of each found paper was then checked in order to include further papers, which could be omitted from the base search strategy due to wrong keywords.

As a first step, the title and abstract of the papers found by the search strategy were evaluated in order to perform a first screening and selection. Successively, the papers to be included in the literature review had to meet the following inclusion criteria: (i) published from 2000 onwards to avoid adding outdated devices or methodologies; (ii) written in English; (iii) conference abstracts were included only if a complete paper on the same topic of the same authors is missing; and, (iv) actual applications with firefighters—papers that speculated on possible applications with FFs without an experimental protocol were excluded. 

### 2.2. Data Extraction and Quality Assessment

The papers that passed the previous selection step and met all the above-mentioned criteria were downloaded and deeply reviewed. Within the aim of providing an overview on the applications of wearable sensors and robotic devices to prevent and monitor work-related diseases, the studies were firstly categorized in two main categories: wearable sensors and robotic devices. Then, information was schematized by a careful reading, obtaining from each paper the following aspects: (i) the aim of the paper; (ii) the number of involved firefighters and their level of expertise; (iii) the used experimental setup; (iv) the implemented experimental protocol; (v) the methodology for data processing and analysis; (vi) the obtained results; (vii) the possible usefulness of the findings. 

In order to provide a quantitative assessment of the paper quality, we used an 18-item questionnaire, similarly to previous literature review [119,120,121,122], taking into account aspects related to the internal, statistical and external validity. The complete checklist is reported in Table 1.

All authors were asked to provide a positive or negative judgement for each item, assigning one or zero points, respectively; then, the final score was obtained by summing each assignment. Only papers that reached a total score equal to or greater than 11 (i.e., obtained a positive score in more than 61% of the items) from the majority of the authors can be considered as “high quality” and are included in the present literature review [119]. 

## 3. Sensor-Based Systems for Physiological Parameter Monitoring

Firefighting is characterized by performing physically demanding tasks in difficult environmental conditions, a combination that results in high levels of physiological stress [47]. In this context, the measurement of the physiological workload of firefighters allows us to understand the factors that contribute to fatigue and to provide a quantitative measure of the physical requirements of a task to reduce risks and improve work performance [64]. 

The difficulty in monitoring and regulating environmental conditions during real firefighting interventions make it difficult to collect data suitable for research purposes [64]. The hazardous environments encountered during real fire activities limit the ability to easily test scenarios to improve safety and effectiveness of interventions or to decrease physiological strain [46]. Although most of the research on firefighters’ physiologic responses has been conducted in laboratories [48,65,67,68,71] and during simulated firefighting activities [4,25,36,46,49,51,54,55,56,62,72,73,74,75,76,80,81,82], few studies are present in literature during real firefighting operations [43,64,69,70,78,83,84].

In the past twenty years, several studies have focused their work on the use of wearable sensors and portable devices to assess physiological variables in firefighters. Wearable sensors are portable, less obtrusive and allow monitoring physiological parameters without interfering with work activities and without constraints imposed by laboratory settings [39,85]. These devices, which can be integrated into clothes and elastic bands or directly attached at different locations of the body, measure several physiological parameters such as heart rate (HR), electrocardiogram (ECG), blood pressure (BP), body temperature (T°), blood oxygen saturation (SpO2) and respiration rate (RR) [63]. 

**Figure 1:**
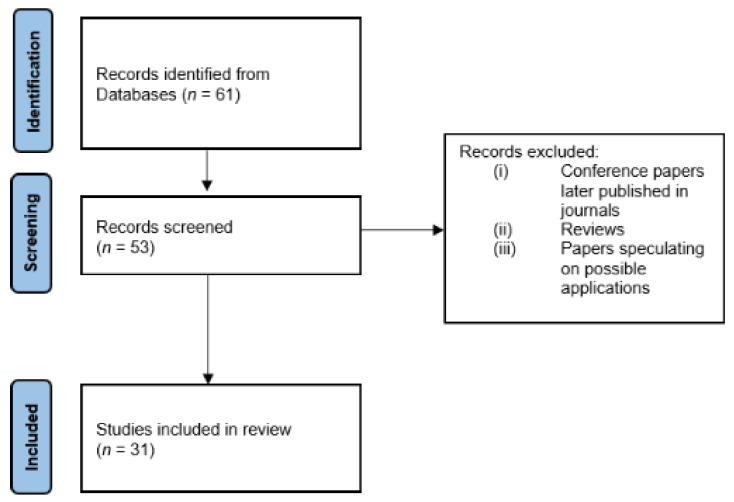
Flow chart of the paper selection for physiological parameter monitoring.

The initial search, conducted according to the steps reported in Methods, yielded 61 papers. After the author, duplicate and language checks, 53 papers remained. We then removed the conference articles that were later published in a journal and excluded all review papers and articles that speculated on possible applications with FFs without an experimental protocol, obtaining 31 papers for thorough reading and analysis. The selection process is shown in Figure 1. 

After analyzing the chosen papers, we described the use of wearable sensors based on the most important physiological parameters to be considered when monitoring the health of firefighters. The distribution of the included papers based on the tested parameter is reported in Table 2. It is worth highlighting that in the majority of case, more than one variable was evaluated in each paper.

### 3.1. Heart Rate

Upon arrival at a fire scene, HR may already be elevated as a stress reaction to the initial fire alarm [66] and continue to be elevated through the working tasks for substantial time periods at levels that are near, and sometimes exceeding, the predicted maximal heart rate [42]. Considering that firefighters are exposed to severe acute and chronic stress leading to cardiovascular problems which are the main cause of line-of-duty deaths [84], the measurement of HR can provide a valuable indication of this cardiovascular strain and help to regulate firefighters’ exertion level [45]. 

Several studies [25,36,46,48,51,55,56,64,69,70,71,74,75,76,77,78,82,83,84,86] have used heart rate monitors to analyze different firefighter HR parameters such as average heart rate (HRavg), peak heart rate (HRPeak), maximum heart rate (HRmax), heart rate of recovery (HRR) and heart rate variability (HRV).

Originally, HR was measured by the count of arterial pulse [37] but the need to measure HR without interrupting physical exercise has led to the development of HR monitors, which are more efficient and accurate tools to obtain a real-time HR measurement. The development of HR monitor devices allows the measurement of real-time HR data using two different types of technology: photoplethysmography (PPG) with optical sensors and the electrical signal from the heart (ECG) [37]. ECG-based HR monitors utilize a chest strap with electrodes to record the electrical activity of the heart [41,79], whereas PPG-based monitors are wrist devices using a light source and a photodetector at the surface of skin to measure the volumetric variations of blood circulation [37,41]. PPG can also be worn on other parts of the body, such as the ear, arm, and forehead.

While both HR monitoring technologies are useful for real-time HR measurement, they present some obstacles. In ECG monitors, HR reading depends on the position of the chest strap electrodes, which must be well positioned close to the heart. In fact, any movement of the chest strap could produce inaccurate HR readings and the use of this procedure could severely limit the flexibility and mobility of users [79]. Contrary, optical noise, skin tone, crossover problem, sensor location and low perfusion can affect HR readings in PPG-based monitors [79]. 

Most of the studies have used ECG-based HR monitors during simulated firefighting activities. In particular, Adams et al. [36] used a heart rate monitor strap fixed around the subject’s torso and connected to a portable unit to measure the peak heart rate HRPeak during simulated firefighting tasks for the development of an occupation-specific training program in cardiac rehabilitation. The results indicated the need for intense, occupation-specific cardiac rehabilitation training that can help firefighters safely return to work after a cardiac event. Del Sal et al. [82] used a heart rate transmitter with an infrared technology attached with an elasticized belt fitted around the chest to measure the HRavg and HRPeak responses to typical activities of firefighters. The data obtained should be considered for the setting-specific training programs that meet the real needs of firefighters in terms of physical fitness. Ensari et al. [46] used a skin-contacting chest strap worn throughout the protocol to measure the HR of 21 firefighters during an intermittent protocol (FAS) that attempts to mimic structural firefighting activity in a laboratory setting. The authors suggested that the proposed protocol can be used to investigate and implement strategies to mitigate metabolic and cardiorespiratory strain of firefighting. Similarly, Johnson et al. [51] used a chest strap heart rate monitor to identify and quantify differences in HRavg and HRmax measures between different firefighter positions in a crew. The findings of the study can be used to develop resistance training and conditioning programs that can better prepare FFs by crew position and reduce the risk of potential cardiovascular incidents while on the job. 

Few studies used portable devices to assess HR even in real work contexts. Meina et al. [84] used sensor belts equipped with a dry-lead ECG to measure real-time HRV during 24 h fire service shifts for detecting psychophysiological stress of 26 firefighters. The results showed that wearable sensors are a valid method of stress level assessment in real-life applications. Similarly, Parker et al. [64] collected the HR using an HR monitor with a chest strap worn against the skin, under clothing, to measure the workload and productivity of the firefighters under real fire conditions. The heart signal receiving unit, integrated with a GPS receiver, was attached to the firefighter’s shoulder strap to record the chest strap signal as a heart rate in beats/minute. The study demonstrated that physiological workload data can be effectively collected during real fires, without compromising fire fighter safety in a complex and unpredictable environment. Rodriguez-Marroyo et al. [83], using an HR monitor to record maximal and mean HR during real wildfire, demonstrated that the type of attack adopted during real wildfire suppression influenced physiological demands in wildland firefighters. These results should be kept in mind when planning programs to improve wildland firefighters’ physical fitness. 

Various studies of Rodrigues et al. [69,70,71] used a wearable monitoring T-shirt (VitalJacket^®^) allowing continuous ECG measurement of firefighters during both work shifts and simulated tasks. Results of the studies have contributed to understanding psychophysiological stress among on-duty FFs through the analysis of HRV parameters that mirrored stressful responses Sebastiao and colleagues [74] used the same T-shirt to monitor the HR of seven firefighters according to different levels of carbon monoxide (CO) exposure during experimental fires. The obtained results allowed the classification of FFs’ exposure to CO levels in relation to monitored HR data.

### 3.2. Body Temperature

The combined effects of strenuous exercise, protective clothing and high external temperatures to which firefighters are often subjected can lead to high levels of thermoregulatory strain [38,59]. The metabolic heat produced by the working muscles, as well as the heat acquired from the external environment, produce an increase in the thermoregulatory strain. Furthermore, the risk of thermal stress experienced by a firefighter’s body is increased by the protective clothing ensembles that limit evaporative heat loss [58]. The inability to lose the increasing amount of trapped body heat leads to peripheral vasodilation to decrease the level of thermal strain, potentially leading to increased cardiovascular demands, which can cause a cardiovascular event or thermal emergency [61]. Furthermore, a high thermal load can result in dehydration, mental confusion and physical fatigue, which can affect firefighter’s performance [44]. Pryor et al. [68] highlighted that is important to monitor the body temperature of firefighters to prevent the onset for heat-related conditions. 

Several studies [40,43,46,48,49,54,55,62,68,72,73,75,77,78,80,82,83,86] have analyzed different body temperature parameters in firefighters such as core body temperature (Tc) and skin temperature (Tsk). Tc is one of the most used parameters for monitoring heat stress in firefighters and represents the temperature of the internal organs of the body, which is normally between 36.5 and 37.4 °C. Heat exhaustion begins to occur when Tc is between 38 °C and 40 °C [59]. Tsk is used in many studies to assess firefighters’ thermal strain, as heat exchanges on the skin surface can both contribute to and challenge thermal homeostasis [87]. 

Available gold standard systems to directly measure Tc are invasive (rectal probe, esophageal probe), uncomfortable and difficult to apply in different work scenarios. Therefore, the ingestible temperature pill represents an alternative and minimally invasive method that allows remote monitoring of Tc [52,57,60]. The telemetry pill contains a thermistor that transmits internal body temperature data to a receiver that collects and records it. Several studies in firefighters [46,48,54,73,77,78,80,83] have used the ingestible pill to measure Tc. Ensari et al. [46] used an ingestible capsule that communicates with a wireless chest worn monitor to measure Tc of 21 firefighters during a simulated firefighting activity station, whereas Savage et al. [73] used a core temperature pill and two portable data logging devices to investigate the relationship between thermal sensation and Tc measurements during active cooling in 49 firefighters. The findings allowed assessing the inadequacy in using thermal sensation as an alternative means of assessing the body’s thermal state. Horn et al. [48] used a Tc pill that communicated with a monitor attached to the belt of 19 career and volunteer firefighters to measure core temperature during different simulated firefighter exercise protocols. The obtained data served to inform researchers and policymakers on the effects of different protocols on physiological responses.

Despite its many advantages, the Tc pill method presents some limitations. The pill must be swallowed at least 4–6 h prior to the measurement which is difficult in real fire conditions because it is unknown when duty will call. Moreover, the temperature pills are difficult to reuse, and their accuracy can be affected by water and food intake [57]. Roosien et al. [72] proposed a new wearable, non-obstructive inner ear thermometer (Cosinuss° GmbH, München, Germany) to monitor changes in Tc in comparison to a temperature pill and a standard inner ear thermometer in 11 firefighters during firefighting simulation tasks. Despite its portability and non-invasive characteristics, the results of the study showed that Cosinuss° is not a valid method for measuring the internal temperature of firefighters while performing their work. Similar results were presented in Pryor et al. [68] where authors examined the agreement between five external thermometers and a gold standard ingestible pill in estimating Tc in 50 firefighters after the heat stress of walking on a treadmill in a heated environment. The outcomes highlighted the inability of external measuring devices to accurately predict Tc in hyperthermic individuals following exertion. Therefore, the authors suggested caution when using any of these temperature estimation techniques.

Moving to the Tsk measurement, the most used quantification method is based on the use of sensors applied directly to the surface of the skin. Horn et al. [49] used skin temperature patches attached to the back of the neck and upper arm that communicated with a monitor attached to the belt of firefighters during different job assignments. The results showed a significant effect of job assignment on both skin temperature measurements, highlighting the need for firefighters to receive rest, recovery and rehabilitation based on the intensity and duration of work. Larsen et al. [55] used skin temperature patches fixed in four sites of the body (middle of the chest, thigh, upper arm, and calf) to assess the accumulated effect of ambient heat on the performance of simulated wildfire tasks over consecutive days. Contrary to the authors’ predictions, the findings showed that work performance across all physical work tasks was unaffected by the heat.

Del Sal et al. [82] used a series of non-invasive biometric sensors embedded in a wireless body monitor worn over the triceps of the right arm to continuously measure heat flux, galvanic skin response (GSR) and Tsk of 13 firefighters during a supervised work test. The results showed that as soon as the firefighters entered the work phase, all variables increased rapidly, as expected. Moreover, it was demonstrated that the body mass of the subjects influences the Tsk during the recovery phase. Cuddy et al. [43] used a monitoring system with an infrared temperature sensor siting against the subject’s skin to measure chest skin temperature of 15 firefighters during live wildland fire suppression. The outcomes of this study highlighted that despite sustaining relatively high chest skin temperatures throughout the work shift, firefighters modulate their work activity to effectively compensate for the environmental conditions and avoid heat-related injuries.

In all the above studies, Tsk was measured with sensors that are wire connected to the measuring device that must be worn on the body. However, a wireless system could be useful for achieving greater comfort and less obstruction of firefighters’ activity. Accordingly, Camera and colleagues [40] proposed epidermal small-size data loggers based on Radio-Frequency Identification technology (RFID), attached to the skin for the continuous monitoring of skin temperature of 10 firefighters, both to investigate the insulating performances of PPE and to supervise the thermal load experienced by firefighters in a minimally invasive way. In particular, for nine firefighters, the three tags were placed with one under the helmet, one on the chest and one on the leg, while for one of the firefighters, the three tags were used to investigate the insulating performance of PPE, with one placed on the chest directly on the skin, the second one on the uniform jacket and the last one in the pocket of the turnout gear coat. The findings revealed that the epidermal dataloggers are reliable, easy to manage and, most importantly, they do not hinder the activity of firefighters. Therefore, the authors suggested improving this technology by adding other sensors (such as humidity, pH) to combine different physiological parameter profiles.

A smart T-shirt developed with the proeTEX project was used in Secco et al. [75], Magenes et al. [86] and Oliveira et al. [62] to measure the body temperature of firefighters during simulated firefighting activities. The authors agree in confirming the efficacy of the ProeTEX wearable system and its capability of real-time and continuous monitoring of the rescuers while they perform highly intense activities in harsh environmental conditions.

### 3.3. Ventilatory Evaluation

Firefighting requires a high level of cardiorespiratory fitness to perform operational tasks safely and effectively [76]. Recommendations require a minimum level of maximum oxygen consumption (VO2max) > 33 mL/kg·min, preferably > 45 mL/kg·min, to successfully complete a rescue protocol standard [66]. In addition, during their activities, firefighters wear a self-contained breathing apparatus (SCBA) to protect themselves from smoke and heat. Although essential for safe operations, SCBA can limit the performance of firefighters by altering ventilatory mechanics and reducing VO2max. The limited air supply contained in the breathing cylinder limits the working range, forcing the firefighter to use different breathing patterns when using the SCBA to conserve breathable air. Intentional hypoventilation while wearing respiratory protection can have physiological consequences for firefighters [50].

Several studies [6,25,36,46,54,56,75,76,81,86] analyzed different respiratory parameters of firefighters including volume of oxygen consumption (VO2), volume of carbon dioxide production (VCO2), breathing volume per minute (VE) and respiratory rate (RR) using portable and wearable devices.

The gold standard for measuring VO2max is indirect calorimetry through the metabolic cart where atmospheric air is inhaled through a mouthpiece and all exhaled air is sent out the mouthpiece into a large base station via a set length of a hose. However, the large size and immobility of the metabolic cart limit its use to specific laboratory settings that restrict range of motion and the types of activities that can be conducted. Therefore, portable devices have been developed to allow the collection of metabolic data in a greater variety of activities [53].

During simulated firefighting activities, Kesler et al. (2018) [48] used a common portable device for breath-by-breath analysis of metabolic data (Cosmed K4b2) to measure the impact of four SCBA configurations and three specific work cycles of varying duration on VO2 and VE of 30 firefighters. Williams-Bell and co-workers [81] used a portable metabolic data collection unit (Cosmed K4b2) with a commercial SCBA facepiece to measure VO2, VCO2, VE and respiratory exchange ratio (RER) during a simulation of a subway system search and rescue. The results from this study revealed the importance of investigating the activities of firefighters while they are breathing through the SCBA device. Ensari et al. [46] used a portable spirometry system, placed in the standard shoulder harness, that was secured with the K4b2 resting on the chest to measure both VO2 and VE during an intermittent protocol (FAS) that attempts to mimic structural firefighting activity in a laboratory setting. The findings demonstrated that the intermittent protocol (FAS) can result in respiratory responses that qualitatively and quantitatively match those from live fire training and response scenarios. Perroni et al. [25] used the same portable metabolimeter (Cosmed K4b2) to measure the VO2 of 20 male professional Italian firefighters and evaluate the energy cost of a simulated firefighting rescue intervention. By providing information on the involvement of metabolic sources during a firefighting intervention, the findings substantiated the need for specific interval training programs for firefighters that aim at increasing their aerobic and anaerobic performances. In the study by Adams et al. [36], 23 healthy male firefighters wore a portable metabolic system (Cosmed K4b2) that allowed the measurement of real-time oxygen consumption (VO2) during a fire and rescue obstacle course that simulated the job demands of a firefighter. The results of the study highlighted the necessity for intense, occupation-specific cardiac rehabilitation training that helps firefighters safely return to work after a cardiac event. The same portable metabolic system was used by Siddal et al. [76] to measure VO2 in 62 operational firefighters. The results of the study allowed the authors to quantify the peak oxygen cost of several simulated firefighting tasks and to derive minimum cardiorespiratory fitness standards for safe and efficient work. Holmer et al. [6] used a portable gas analysis equipment (Metamax, Cortex, Germany) to measure the energetic and respiratory responses of 15 male professional firefighters to a simulated firefighting exercise in a training house. The results demonstrated that work tasks associated with firefighting require considerable energetic demands, in many conditions close to the maximal capacity of the individual.

RR, also known as respiratory frequency (Rf), is another respiratory parameter analyzed in firefighters, which represents the number of breaths per minute, or the number of respiratory cycles completed in 1 min. Monitoring of RR during firefighting activities is of great importance because RR is sensitive to cognitive load, emotional stress, environmental challenges, pain and body temperature [123]. Marcel-Millet et al. [56] used a wearable suit to measure the breathing rate (BR) assessing the cyclic motion of the chest through two strain bands located on the chest and the abdomen. The study aimed to assess the effect of wearing a breathing apparatus during a simulated rescue intervention on psycho-physiological responses and parasympathetic reactivation of firefighters. Based on the results obtained, rescue interventions seem to lead to high physiological stress (i.e., BR values), executive function perturbations (i.e., accuracy) and important post-exercise vagal perturbation. In addition, the study showed that SCBA increased psychophysiological perturbations. Similarly, Secco et al. [75] and Magenes et al. [86] used a smart T-shirt developed with proeTEX project to measure BR in six and 13 professional Italian firefighters, respectively, during field trials through a piezo-electric transducer that generates signals related to the chest movement.

### 3.4. Multi-Sensors Monitoring System

The use of specific monitoring systems for each parameter may not be a practical and ergonomic solution for continuous assessment during firefighting. Therefore, a wearable multi-sensor monitoring system can be a viable solution for multi-parameter monitoring [85]. Currently, several multisensory monitoring systems are commercially available; however, only few studies have used a multi-sensor monitoring system to simultaneously monitor several physiological parameters in firefighters.

The Equivital EQ02 LifeMonitor (Hidalgo; Cambridge, UK) is a wearable monitoring system for collecting multiple physiological parameters such as HR, HRV, RR, ECG, Tsk and Tc. It consists of an electronic module with a Lycra sensor belt, an ingestible Tc pill, multiple dermal patches (up to seven) and two ancillary sensors (galvanic skin response sensor and oxygen saturation sensor). Few studies used the Equivital EQ02 LifeMonitor to assess different physiological parameters in firefighters. Horn et al. [53] used the Equivital LifeMonitor EQ02 to measure both the HR and Tc of 19 firefighters during three different exercise protocols, whereas Kesler et al. [54] used it to measure HR and collect data transmitted by the ingested Tc pill of 30 firefighters during seven trials using different SCBA. 

The SenseWear PRO2 Armband (SP2) is a metabolic Holter device worn over the triceps that uses a series of noninvasive biometric sensors to continuously measure different physiological parameters such as heat flux, GSR, Tsk, energy expenditure as metabolic equivalent of task (MET) and motion determined from a two-axis accelerometer. Del Sal et al. [82] used the SP2 on 13 healthy military firefighters during typical firefighting activities.

The research progress of wearable systems for health monitoring has led to the development of monitors embedded in wearable garments. Secco and colleagues [75] used a smart T-shirt developed with the proeTEX project to measure the HR, BR and body temperature (BT) in six professional Italian firefighters during both laboratory and field tests in harsh and uncontrollable conditions. The smart T-shirt aims to monitor the physiological parameters of the firefighters thanks to a set of sensors incorporated in an elastic region in direct contact with the subject’s skin. In addition, the T-shirt incorporates an electrical part, supporting the sensors, located within another textile band that surrounds the chest, a rechargeable battery to power the system and a ZigBee wireless communication module to transmit all data of the sensors. The same T-shirt was used by Magenes et al. [86] to assess HR, BR and BT during field test on 13 firefighters. Marcel-Millet et al. [56] used a wearable suit (Hexoskin^®^ Carré Technologies Inc., Montreal, QC, Canada) to measure BR and HR of 34 firefighters during simulated rescue intervention. The Hexoskin shirt is a wearable device that measures multiple physiological functions simultaneously. The cardiac measurements are made using three cardiac electrodes embedded in the shirt at sternum level and at abdominal level to produce a one-lead ECG. The respiratory measurements are measured by two magnetic sensors located anteriorly at sternum level and along the abdominal area that measure the shape of the body while breathing.

## 4. Sensor-Based Systems for Physical Parameter Monitoring

The high-demand tasks that firefighters are asked to perform daily lead to a high risk of work-related physical injury [29]. For this reason, the analysis of the physical parameters, such as mobility and balance, is an always more widespread approach for (i) understanding the musculoskeletal characteristics of professional firefighters in order to set mandatory requirements for the recruitment, (ii) identifying potential injury factors to monitor and (iii) improving the design of PPEs that strongly affect the physical performance of a firefighter. 

As for the physiological parameters, the environmental conditions in which firefighters usually work make difficult the real-time evaluation of such parameters and the majority of the studies deal with the physical evaluation in simulated controlled laboratory-based conditions. For the same reason, the spread of wearable sensors is still limited to only inertial units, whereas optoelectronic systems and force platforms are the most used sensor-based systems. 

**Figure 2:**
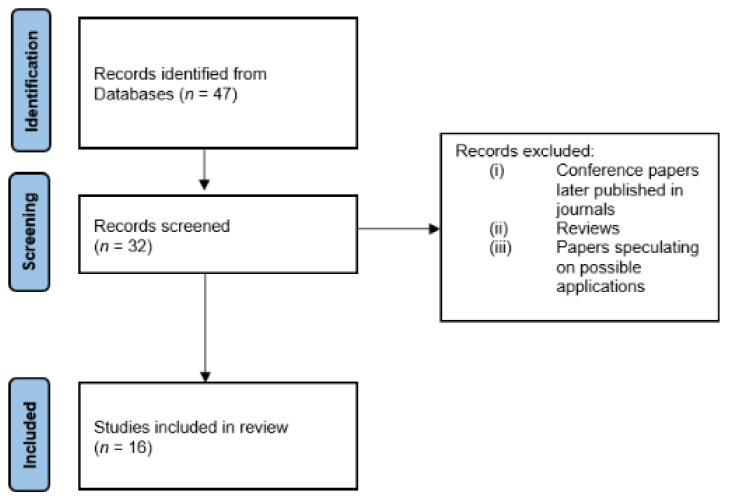
Flow chart of the paper selection for physical parameter monitoring.

The initial search, conducted according to the steps reported in Section 2, yielded 47 papers. After the author, duplicate and language checks, 32 papers remained. We then removed the conference articles that were later published in a journal and excluded all review papers and articles that speculated on possible applications with FFs without an experimental protocol, obtaining 16 papers for thorough reading and analysis. The selection process is shown in Figure 2. 

After analyzing, we categorized papers based on three main topics regarding physical performance: mobility, posture and muscle activity. The distribution of the included papers based on the three categories is reported in Table 3. It is worth noting that muscle activity was always evaluated combined with a mobility analysis. 

### 4.1. Mobility and Muscle Activity

It is well established that the main negative effect on the mobility of firefighters is due to PPEs that lead to a decrease in physical performance [29]. Many different approaches have been proposed to quantitatively measure the mobility of firefighters during typical activities and the majority focused the analysis on the evaluation of the range of motion (ROM) [88,93,95,96,98,101,102,103,105].

Coca et al. [88] implemented an experimental protocol including eight subjects, asking them to perform static, dynamic and job-related tasks while wearing a standard fire fighter ensemble (SE) or regular light clothing. Specifically, static ROM tasks consisted of measuring flexion/extension and abduction of elbow, shoulder, neck, hip, knee, ankle and wrist joints; as for dynamic ROM tasks, they included kneel and rise, seated squats and step-ups. In addition, subjects had to perform ensemble donning/doffing, one-arm search, ladder pickup, crawling over and under objects, mannequin drag and solid object lift to simulate job-related tasks. The measures were conducted using a goniometer and torso bend device. The findings revealed that the tasks performed with SE are characterized by a significant reduction in shoulder flexion, cervical rotation and flexion, as well as a decrement in trunk lateral flexion. The comparison among clothing variations was carried out also by Orr et al. [102], who tested eight firefighters when performing several job-related tasks, as vertical jump, stair and ladder climbing, low crawl and the tasks included in the Functional Movement Screen (FMS). After each of the assessed tasks, the participants were asked to rate the perceived impact of their clothing on mobility and comfort. The results confirmed that heavy clothing are perceived by firefighters as a greater discomfort and increased injury risk, especially related to the lower limbs. Similarly, the lower limb range of motion was evaluated by Park et al. [93] by enrolling twelve firefighters. Tests included a ten-meter walking routine at self-preferred pace in five different garment conditions: (i) T-shirt and shorts with running shoes; (ii) turnout coat and pants with running shoes; (iii) adding an SCBA air tank of 8.1 to condition (ii); (iv) using rubber boots in substitution of running shoes; (v) the same condition as (iii) but using leather boots. Inertial-based motion capture system was used to compute joint angles of lower limbs and, successively, to compute the range of motion. All the movements in sagittal and transversal plane were subjected to a reduction when wearing the turnout ensemble and SCBA, whereas the addition of rubber boots caused a reduction in the ROM for anterior-posterior movements at the ankle and foot level. Conversely, an increment in the ROM for medio-lateral movements of the foot was observed. The effects of the boot were also more significant when considering female firefighters. The findings of the present study can be considered when seeking to identify the specifications for ergonomic boot design. The reduction in ankle range of motion caused by the use of boots was also confirmed by Vu et al. [95], who evaluated the lumbar and lower limb biomechanics of 20 professional male urban firefighters when performing drop jump task. The analysis was conducted by using optoelectronic system in a laboratory-based environment. The authors correlated the decrement in ankle movements with the increment in lumbar injury risk, assessing that the proper design of boots is a fundamental requirement for reducing this risk. The influence of the boot height was investigated by [98] in order to understand if the fixed boot height can lead to a greater worsening of lower limb mobility in shorter firefighters. Experimental protocol involved 21 participants performing walking, duckwalking and ladder climbing tasks. Data were acquired through inertial sensor units for lower limb kinematics and a 3D body scanning for the upper body. As expected, shorter firefighters revealed a significant decrement in mobility, mainly due to the reduced clearance between knee and the top of the height-fixed boots. In the same paper, shorter firefighters were also found having reduced upper body mobility due to the SBCA, especially for neck extension and lumbopelvic flexion. The outcomes of this study highlighted the necessity to take into account the human factors and anthropometry data when designing fire gear. The comparison between gender regarding the effects induced by donning a structural turnout ensemble was accomplished by McQuerry [105]. Specifically, ten male and six female firefighters were tested in static ROM and standing/sitting tasks. Body scanner systems and electrogoniometers were used to gather quantitative data related to the subject mobility. The findings allowed assessing that mobility was significantly reduced when donning the SE and the gender effect was mainly found related to the trunk and shoulder flexion. Such results should be exploited in order to develop female-specific structural turnout gear sizing systems.

Another important factor deeply studied in literature regarding the mobility is the foot clearance when traversing stairs [91,94]; in fact, slip, trip and fall have been demonstrated to be among the main causes of moderate to severe firefighters’ injuries [124]. In this context, Kesler et al. [91] assessed the effects induced by fatigue and load carriage on foot clearance when traversing stairs. The experimental protocol involved 24 firefighters and it consisted of a preliminary phase and a motion task. As for the preliminary phase, the fatigue was induced by simulating firefighting tasks—i.e., climbing stairs, advancing a weighted hose line, searching a room and pulling down a ceiling—for 14 minutes in both environmental chambers and in a burning building. Successively, the participants were asked to perform a motion task consisting of (i) traversing a short stairway, (ii) crossing over a three-step wooden-frame stairway and (iii) ascending one side and descending the opposite always facing forward. An optoelectronic system was used to measure the landing and passing foot clearance by using passive reflective markers on heel, first metatarsal, fifth metatarsal and the tip of the boot. In average, a decrease in clearance was found during ascent and an increase was found during descent. These significant changes in clearance may be correlated to an increment in risk injuries due to tripping over stairs during ascent or slipping during descent. The effects induced by different boots was, instead, evaluated in [94]. The authors tested 30 firefighters wearing both rubber and leather boots when performing three minutes of stair climbing and a slip trial without the knowledge of the slippery floor. Optoelectronic system was used to evaluate the heel slip velocity in both vertical and horizontal directions and the heel clearance. The results suggested that rubber boots elicit greater slip severity both in expected and unexpected slips compared to those made of leather. Such findings should be taken into account when deciding the PPE usage according to the specific activities to carry out. 

A more complete analysis of the mobility, seen as combination of kinematic and muscle activity data, has been proposed by Son and colleagues [103]. The authors evaluated the effects of various clothing, in terms of material and shapes, considering both range of motion and electromyography. The study involved eight participants performing five motor tasks: (i) shoulder flexion/extension; (ii) shoulder adduction/abduction; (iii) shoulder rotation; (iv) trunk flexion/extension; (v) hip adduction/abduction. Kinematic data were recorded by using an optoelectronic system and 26 passive markers. The muscle activity was recorded by means of surface EMG probes related to middle deltoids, biceps brachialis, triceps, rectus femoris and semitendinosus. ROM and maximum voluntary contraction (MVC) have been assumed as synthetic indices. The results showed an increment in the range of motion if the standard uniform is substituted by stretch-wear and compression-wear. The same materials were not proven to guarantee the muscle performance. The findings proposed in this paper should be considered for the proper selection of the material for the realization of firefighters’ clothing.

Summarizing, it is clear how all the discussed papers, and more in general, all the papers investigating the mobility, mainly deal with understanding the effects induced by mandatory equipment of firefighters in order to provide useful guidelines for the most appropriate design of PPE, ranging from boots to SCBA. 

### 4.2. Posture

The loss of balance represents one of the main causes of falls and injuries in firefighters [124]. In this context, the abilities of firefighters to maintain equilibrium when performing different tasks have been deeply investigated in literature [89,90,92,99,100,104,106]. Among them, almost all studies investigated the effects of personal protective equipment given the correlation between their weight and firefighters’ stability [90,92,99,100,104,125].

The effects induced by the firefighters’ equipment were assessed by Park et al. [92] who enrolled 12 participants in an experimental procedure consisting of a 10 m walking task at a self-selected speed. The participants were tested in five different garment conditions by adding external apparatus, starting from normal wear and building up to the firefighters’ complete equipment, including boots and SCBA. An in-shoe plantar pressure sensor was used to calculate the center of plantar pressure. The addition of essential equipment led to a decrease in AP and ML excursion of center of plantar pressure, as well as its velocity decrement. Such decrements were more evident in case of leather boots compared to the rubber ones. A similar approach was proposed by Brown and colleagues [99], who aimed at revealing possible factors in fall-related injuries and identifying strategies to reduce occupational risk. Static and dynamic tests were conducted with 21 municipal firefighters and a Biodex Balance System (BBS) was used to assess firefighters’ ability in maintaining equilibrium. Outcomes showed that the use of turnout ensemble, SCBA and face mask negatively affected dynamic balance, with the greater impact due to the face mask, which influences the visual condition. The authors suggested the development of specific training programs for the promotion of occupational safety. All the directions in the plane have been found affected by PPE also in the study conducted by Games et al. [100], when 40 male firefighters performed the lower quarter Y Balance Test. Similarly, Wiszomirska and colleagues investigated the risk factors leading to falling in 117 firefighters. Participants were tested by means of the BBS and the fall risk test, analyzing the overall stability, the anterior-posterior stability, the medial-lateral stability and the fall risk index. The effects on these parameters caused by age, clothing and visual conditions were assessed. As the main finding, the authors demonstrated that bunker gear worn without an SCBA and a face mask did not have effect on balance performance; conversely, the age and the visual conditions had a significant impact on postural stability. It is thus evident that special balance training, especially in poor visibility conditions, should be introduced to prevent injuries. Focusing only on SCBA, Hur et al. [90] evaluated the effect induced by bottle designs, differing in mass and size, on postural control. Experimental protocol involved 24 firefighters, asking them to stand in a comfortable stance on a force platform in both open and closed eyes conditions. Tasks were also performed in unperturbed and perturbed mode, with the last consisted of a mild impulsive backward tug applied to the subject’s waist through a seat belt mechanism. Data related to the center of pressure and its components in AP and ML directions were computed. The procedure was repeated by testing four different SCBA: aluminum bottle, carbon fiber bottle, fiberglass bottle and a home-made bottle. The authors assessed that heavy bottles only increased the postural sway in medio-lateral direction; instead, the AP direction was modified only in case of vision absence. These outcomes should be exploited for the design of an SCBA characterized by reduced weight, smaller heights and center of mass closer to the body of the firefighter. 

Regardless of the impact of the equipment, it is clear how the physical characteristics of the firefighters have a significant correlation with the balance abilities. Davis et al. [89] aimed to determine the effects induced by an excessive body weight on postural balance. Experimental protocol was conducted with thirteen firefighters, of which six classified as obese and seven classified as overweight. The center of pressure was monitored through a portable postural balance measurement system composed of a force platform system (Accusway Plus). Participants were asked to perform static balance test, i.e., standing position, on both open and closed eyes and a dynamic reach task. Obese firefighters showed less postural sway, with a reduction of 26% when standing and 18% during reach task when compared with overweight firefighters. In the same context, Marciniak and colleagues [106] sought to understand the relationship between postural ability and physical fitness variables in firefighters. A total of 35 firefighter recruits were enrolled in the study, measuring the outcomes of Y-balance tests, body mass index, body-fat percentage, fat free mass, aerobic capacity in terms of VO2max, stair climbing, and upper and lower-body strength through bench and squat press and Fusionetics Movement Efficiency Screen. The outcomes of the dynamic test were found strongly correlated only with the body mass index, movement quality and lower-body strength; specifically, greater balance ability was associated with lower body mass index, greater functional movement and greater lower-body strength. The authors suggested that these variables should be incorporated into balance training programs. 

Summarizing the results related to sensor-based systems considering both physiological and physical parameters, still few studies have performed evaluations in real settings, limiting the applications to laboratory-based scenarios. Future studies should be conducted to understand the effective feasibility to use only wearable sensors to monitor firefighter performance during work-related activities, enhancing the possibility to measure realistic parameters for reducing work-related disorders. In addition, a further step forward could be represented by the introduction of smart wearable textiles that can be embedded into the firefighters’ PPE for the realization of “futuristic equipment”. Such equipment could be used not only to gather useful information on firefighters’ performance, but also in active mode by sending immediate feedback to the firefighters in case of a hazardous situation, such as wrong posture, excessive joint mobility or inadequate physiological values.

## 5. Future Perspectives: Robotic and Assistive Devices

The specificity of robotic devices designed for firefighters is related to their particularly dangerous working conditions. Firefighters are usually asked to wear a set of fire-fighting tools and equipment weighing about 20 kg, they walk inside smoky environments using insulating breathing apparatuses, they control high-performance hoses when extinguishing fires, overcoming the significant reactive forces of the formed fire-extinguishing jets [114]. To prolong action time of firefighters, it is necessary to enhance the performance in terms of endurance, mobility and load capacity. Hence, equipment for helping carry loads is a great advantage both to hold out and remain effective in the effort made.

The main issue in developing such robotic devices is related to the working environment of firefighters. Fire emergencies are characterized by very high temperatures that lead to break-ups in mechanical components [126].

**Figure 3:**
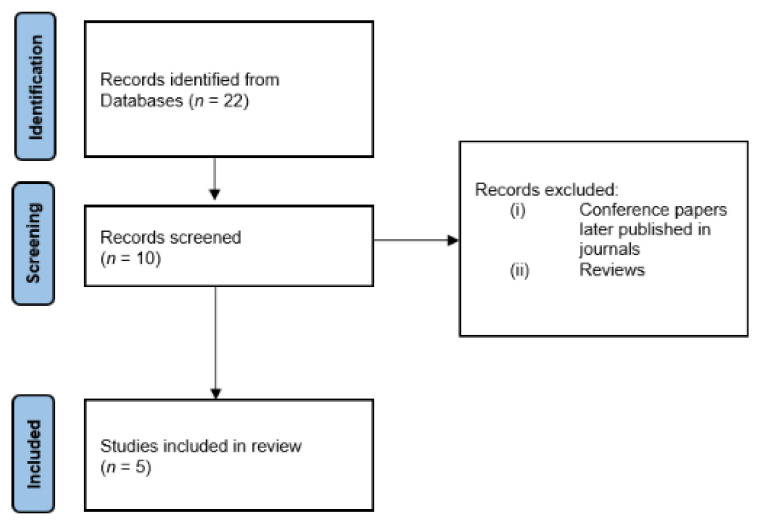
Flow chart of the paper selection for robotic devices.

The initial literature search, conducted according to the steps reported in Section 2, yielded 22 papers. After the author, duplicate and language checks, 10 papers remained. We have then removed the conference articles that were later published in a journal and excluded all review, obtaining 5 papers for thorough reading and analysis. It is worth noticing that the use of robotic devices in this field is still an untapped issue; for this reason we decided to include also possible speculation of exoskeletons/assistive devices scalable for firefighters. The selection process is shown in Figure 3. 

After analyzing, we categorized papers in two main groups based on the typology of developed robots. The identified groups are exoskeletons and assistive devices. The distribution of the included papers based on the two categories is reported in Table 4. 

### 5.1. Exoskeletons

Exoskeletons are robotic devices directly worn by the users. They are equipped with a series of sensors and actuators usually to augment users’ movement and strength and, thus, their physical performances. Fire exoskeleton development is a subcategory of the industrial exoskeleton development research area.

Even if no papers have been published describing the development of exoskeleton specifically designed for firefighters, some works describe the development of exoskeletons for workers in extremely high-risk environments (for example, in presence of nuclear, chemical, marine dangers).

The main issues that must be addressed in developing such an exoskeleton regard design and control. In [111], some key factors in developing industrial exoskeletons are reported:

- Comfort: Two main aspects affect the comfortability of an exoskeleton, the weight and the body attachments. Extending the exoskeleton to the ground counters the weight but increases the design complexity, thereby reducing the transparency of the system.
- Transparency: The system transparency is dictated by the kinematics of the design and the back-drivability of the actuation.
- Intuitive control: The system should follow the movement of the operator, providing the correct level of assistive forces.

Exoskeletons are usually designed for upper or lower body. Lower body exoskeletons usually have a simpler design compared to upper body exoskeletons thanks to simpler kinematics (less degrees of freedom). From a mechanical point of view, lower limb exoskeletons must adapt to user height to allow a good level of transparency. In [116], a power assist walking support system has been developed. The system has to be attached directly to the bilateral sides of the human body and it has four links (hip, thigh, lower leg and foot) and five rotational degrees of freedom on each leg (three on the hip, one on the knee and one on the ankle). Every joint range is also limited to prevent their hyperextension. To avoid the motion collision between the exoskeleton frame and the user, the designed joint axes and human joint axes must be coaxial. The actuators are DC servo motors designed to generate enough assistive force on the hip joint and the knee joint. The exoskeleton has several sensors to quantify the human–robot interaction. Two dimension force sensors are placed on the exoskeleton, directly in contact with human legs through bundles, to measure the motion difference between the human and the exoskeleton. Exoskeleton control is based on a mass-spring-viscidity model (force and velocity control) that analyzes force sensor output and encoder values to assess user motion intention using sensor fusion techniques, and to provide the right torque to servo motors. 

Regarding upper body exoskeletons, in [115], a robotic arm specifically designed for soldiers was developed. This concept can be replicated for firefighters because the exoskeleton has been designed to augment the user’s ability to maneuver heavy tools such as fire-extinguishing jets. The exoskeleton is attached to the user’s upper body via a passive link at the shoulder, elbow and wrist. Mechanically, the exoskeleton behaves like two four-bar mechanisms attached to one another. The shoulder’s elevator DOF and the elbow are actuated while shoulder azimuth and wrist are passive. Each joint has an encoder to measure the actual angular position. The control system analyzes the user’s angular position to define the desired shoulder and elbow torques.

### 5.2. Assistive Devices

Although exoskeletons increase human capabilities, they do not increase the safety level of the user. This is especially critical in hazardous situations as during firefighting. For these reasons, many studies focused on the development of new robotic devices that can substitute firefighters to protect them from exposure to hazardous substances, environments and/or physical agents. This strategy can be generally applied in hazardous industrial applications such as nuclear or chemical industries. Only few papers describe the development of assistive devices specially designed for firefighters.

In [113], an autonomous firefighting robotic arm is introduced. The developed robot works similar to a fireman’s hand in firefighting scenarios. The described firefighting robotic arm is a remote-controlled robot which can be used for firefighting effectively. It consists of a microcontroller, wheels for the drive, motor driver IC, DC motors, servo motors, power, a ZigBee communication device, a printed circuit board and an effective firefighting media such as water. The paper describes the initial design and fabrication. No real experiments have been conducted. Even if most of the published papers describe the development of autonomous solutions, there are still limitations on capabilities of robots because humans work better on certain tasks than robots. Thus, collective intelligence, which allows interaction between a human and a robot (as for the exoskeletons), is desired to produce the most efficient output with minimum resources. In [112], the authors analyzed the problem of communicating with such robots. This problem can be critical, especially when robots have been manufactured in a completely different part of the world. The authors suggested a solution to develop a natural language interface between firefighter and robot so they can act as a team during a dangerous and potentially lethal fire scenario, and they can work using an interface similar to that between two human firefighters. The authors used Ontological Semantic Technology (OST) to address meanings in an easily comprehensible way as a human does. In order to implement ontology-based communication with different languages, Korean and English were used for this particular study. The results showed no significant differences among different types of languages as well as translations, as both the accuracy and the acceptability of the commands are similar. Although the average accuracy on the general commands is mediocre, both results on the acceptability are high. This indicates that a successful integration, through natural language, of the firefighter and the robot is very reasonable.

Even if only few papers have been identified, the development of robotic devices for hazardous operations could have an important role in the next years to (i) reduce workers’ physical and physiological stress and (ii) increase working environment safety. The design approach and the kind of technology solution proposed can be applied to other hazardous scenarios. These next-generation industrial exoskeletons and collaborative robotic systems can address the emerging challenges in industrial workers’ health and safety.

## 6. Conclusions

The use of sensor-based systems for monitoring both physiological and physical parameters achieved popularity during firefighter activities with the main aim of reducing work-related diseases. The present literature review indicates that physiological parameters have been evaluated deeper than the physical ones, especially regarding the heart rate, the body temperature and the ventilatory evaluation. Among physical parameters, mobility of both upper and lower limbs, as well as the evaluation of balance ability represent the main fields of investigation. The advent of wearable and unobtrusive sensors has allowed measuring the parameters of interest during real-life activities, as well. The examined papers reveal that the use of personal equipment represents the main problem leading to a worsening of the physiological and physical performance of firefighters, indicating that the well-conceived design of such equipment is essential to avoid work-related diseases. 

On the other hand, the use of robotic devices is still uncertain, as indicated by the few found papers. However, it is more than credible that exoskeletons and assistive devices will represent the future direction in hazardous working activities, such as those required of firefighters.

## Figures and Tables

**Table 1:** Quality assessment for internal (IV), external (EV) and statistical (SV) validity.

Criteria	Type of Validity
Aim of the work	
Description of a specific, clearly stated purpose	IV
The research question is scientifically relevant	EV
Inclusion criteria (selection bias)	
Description of inclusion and exclusion criteria	IV-EV
Inclusion and exclusion criteria are the same for all tested groups	IV
Inclusion and exclusion criteria reflect the general population	EV
Data collection (performance bias)	
Data collection is clearly described and reliable	IV-EV
Same data collection method used for all the subjects	IV
The used setup is wearable	EV
Data loss (attrition bias)	
Different data loss between groups	IV
Data loss < 20%	EV
Outcome (detection bias)	
Outcomes allow tangible applications	EV
Outcomes are the same for all the subjects	IV
Data presentation	
Frequencies of most important outcome measures	IV
Presentation of the data is sufficient to assess the adequacy of the analyses	IV
Statistical approach	
Appropriate statistical analysis techniques	SV
Clearly state the statistical test used	SV
State and reference the analytical software used	SV
At least five tested subjects	SV

**Table 2:** Selected paper distribution based on the specific parameter.

Parameter	Number of Papers	References
Heart rate	18	[25,36,46,48,51,56,64,69,70,71,74,75,76,77,78,82,84,86]
Body temperature	18	[40,43,46,48,49,54,55,62,68,72,73,75,77,78,80,82,83,86]
Ventilatory evaluation	10	[6,25,36,46,54,56,75,76,81,86]

**Table 3:** Selected paper distribution based on the physical categories.

Categories	Number of Papers	References
Mobility	9	[88,91,93,94,95,98,102,103,105]
Posture	7	[89,90,92,99,100,104,106]
Muscle activity	1	[103]

**Table 4:** Selected paper distribution based on the type of robot.

Categories	Number of Papers	References
Exoskeletons	3	[111,115,116]
Assistive devices	2	[112,113]

## Data Availability

All relevant data are within the manuscript.
